# Low-dose aspirin and racial disparities in spontaneous preterm delivery in low-risk individuals

**DOI:** 10.1016/j.xagr.2023.100273

**Published:** 2023-10-05

**Authors:** Veronica A. Kane, Maria Andrikopoulou, Clara Bertozzi-Villa, Joseph Mims, Kelsey Pinson, Cynthia Gyamfi-Bannerman

**Affiliations:** 1 Columbia University Vagelos College of Physicians and Surgeons, New York, NY (Ms Kane); 2 Division of Maternal-Fetal Medicine, Department of Obstetrics and Gynecology, Columbia University Irving Medical Center, New York, NY (Drs Andrikopoulou and Bertozzi-Villa); 3 Department of Obstetrics and Gynecology and Women's Health, Montefiore Medical Center, Bronx, NY (Dr Bertozzi-Villa); 4 Division of Maternal-Fetal Medicine, Department of Obstetrics, Gynecology, and Reproductive Sciences, University of California San Diego Health, La Jolla, CA (Drs Mims, Pinson, and Gyamfi-Bannerman)

**Keywords:** African American, Black, health equity, low-dose aspirin, nulliparous, placental disease, platelet aggregation, preeclampsia, preterm birth, racial and ethnic disparities, spontaneous preterm birth, White

## Abstract

**BACKGROUND:**

Preterm birth is a leading cause of perinatal morbidity and mortality. There are significant racial disparities in the rates of preterm delivery in the United States, with Black individuals at disproportionately higher risk than their White counterparts. Although low-dose aspirin is currently under investigation for reducing the rates of preterm delivery, limited data are available on how the use of low-dose aspirin might affect racial and ethnic disparities in the rates of preterm delivery.

**OBJECTIVE:**

Our group and others have shown that low-dose aspirin decreases spontaneous preterm delivery in low-risk parturients. This study aimed to examine whether the relationship between low-dose aspirin and the risk of spontaneous preterm delivery is modified by race and ethnicity.

**STUDY DESIGN:**

This was a secondary analysis of a randomized clinical trial examining low-dose aspirin for preeclampsia prevention in low-risk nulliparous individuals. The parent trial defined low risk as the absence of preexisting hypertension or other medical comorbidities. Participants received 60-mg aspirin or placebo between 13 and 25 weeks of gestation. Here, multiple pregnancies, fetal anomalies, terminations or abortions at <20 weeks of gestation, and participants with previous miscarriages were excluded. Our exposure, race and ethnicity, was self-reported in the parent trial and categorized as non-Hispanic White, Hispanic, non-Hispanic Black, and other. The primary outcome was spontaneous preterm delivery at <34 weeks of gestation; the secondary outcomes included spontaneous preterm delivery at <37 weeks of gestation and all preterm deliveries at <34 and <37 weeks of gestation. Fit logistic regression models were used to examine how the use of low-dose aspirin modified the relationship between race and ethnicity and preterm delivery, adjusting for confounders. Furthermore, sensitivity analyses were performed to compare the rates of preterm delivery by race and ethnicity.

**RESULTS:**

Of note, 2528 of 3171 parent study participants were included in this analysis. Of the participants, 425 (16.8%) were White, 819 (32.4%) were Hispanic, 1265 (50%) were Black, and 19 (0.8%) were other. The baseline characteristics differed among racial and ethnic groups, including maternal age, body mass index, education level, marital status, tobacco and alcohol use, and pregnancy loss. The rate of spontaneous preterm delivery at <34 weeks of gestation was significantly higher in Black participants (2.8%) than in White (1.2%) and Hispanic (1.2%) participants (*P*=.04). Logistical regression analysis showed that Black race was no longer an independent risk factor for spontaneous preterm delivery at <34 weeks of gestation when controlling for low-dose aspirin (adjusted odds ratio, 1.71; 95% confidence interval, 0.67–4.40). A similar pattern was found for spontaneous preterm delivery at <37 weeks of gestation and preterm delivery at <34 and <37 weeks of gestation. In our sensitivity analyses, spontaneous preterm delivery at <34 weeks of gestation differed by race and ethnicity in the placebo group (*P*=.01) but did not differ in the low-dose aspirin group (*P*=.90).

**CONCLUSION:**

The use of low-dose aspirin mitigated racial disparities in spontaneous preterm delivery at <34 weeks of gestation. Additional investigation is warranted to assess the reproducibility of our findings.

**AJOG Global Reports at a Glance**

**Why was this study conducted?**

This study aimed to examine whether the relationship between low-dose aspirin (LDA) and the risk of spontaneous preterm delivery in low-risk nulliparous individuals is modified by race.

**Key findings**

The use of LDA mitigated racial disparities in the rates of spontaneous preterm delivery at <34 weeks of gestation in low-risk nulliparous individuals.

**What does this add to what is known?**

Given the significant burden of preterm delivery for African American individuals in the United States, our findings represent a promising area of research to promote more equitable maternal and fetal health outcomes.

## Introduction

Preterm birth is the leading cause of perinatal morbidity and mortality and the most common cause of death under the age of 5 years worldwide.^1^^,^^2^ Of note, 70% of preterm deliveries occur spontaneously.^3^ In addition to its significant costs to the healthcare system, estimated in 2016 to reach $25.2 billion in the United States,^4^ preterm delivery (PTD) is associated with substantial psychosocial consequences to families of preterm infants.^5^

There are significant racial disparities in the rates of PTD in the United States that are well described.^2^^,^^6^, ^7^, ^8^, ^9^, ^10^ In 2020, the Centers for Disease Control and Prevention (CDC) estimated that Black individuals had a 50% higher rate of PTD than people who were White or Hispanic: a rate of 14.4% vs 9.1% and 10.0%, respectively.^11^ Although the rate of preterm birth declined significantly between 2019 and 2020 for non-Hispanic White and Hispanic individuals, there was no significant decline in the rates for non-Hispanic Black individuals, further contributing to this disparity.^11^ Of note, 54% of neonatal mortality for non-Hispanic Black deliveries is attributable to preterm birth.^10^ Despite not being fully understood, proposed mechanisms underlying these disparities include systematic racism with downstream effects on environmental and other sociodemographic factors that predispose to PTD.^7^^,^^8^^,^^12^, ^13^, ^14^

The use of low-dose aspirin (LDA) has been studied as a prevention method for both spontaneous and medically indicated PTD. Previous studies have shown that the use of LDA reduces spontaneous PTD (sPTD) in those with a history of pregnancy loss^15^ and those at risk of preeclampsia.^16^ Although a recent, small randomized control trial (RCT) found a modest, but nonsignificant, decrease in sPTD for those on LDA with a history of sPTD,^17^ a subsequent large cohort study demonstrated that the use of LDA significantly reduced the rates of recurrent sPTD.^18^ Furthermore, our group has shown that the use of LDA is associated with a reduction of sPTD in low-risk nulliparous individuals.^19^ Our findings were confirmed by a randomized, double-blind, placebo-controlled trial, which showed that the use of LDA reduced the risk of PTD in nulliparous participants with a singleton pregnancy in a large international sample.^20^ However, a search of PubMed for articles on PTD and LDA yielded no other studies describing the effect of LDA on racial disparities in the rates of sPTD. This study aimed to examine the relationship among LDA, racial and ethnic background, and the risk of sPTD in low-risk women. We hypothesized that the use of LDA would decrease the rate of sPTD within racial and ethnic groups and possibly mitigate racial disparities in sPTD.

## Materials and Methods

This was a secondary analysis of the *Eunice Kennedy Shriver* National Institute of Child Health and Human Development Network of the Maternal-Fetal Medicine Units’ randomized placebo-controlled trial of LDA (60 mg) for the prevention of preeclampsia in low-risk individuals. The parent trial included participants at 13 to 25 weeks of gestation without chronic conditions, including hypertension; diabetes mellitus; seizures; or renal, heart, or collagen or vascular disease. Complete details of the study design and methods have been previously reported.^21^ The study data are publicly available and deidentified. Therefore, this study was exempt from institutional review board review at our institution.

Here, we additionally excluded the following: multiple pregnancies, pregnancies complicated by fetal anomaly, and antepartum stillbirth. In addition, individuals without outcome data, individuals with spontaneous abortion at <20 weeks of gestation, and individuals who terminated pregnancies were excluded. Our primary exposure was self-reported race and ethnicity, initially characterized by the parent trial into the following categories: White, non-Hispanic; White, Hispanic; African American, non-Hispanic; African American, Hispanic; American Indian or Alaskan Native; Oriental; Asian Indian; and Asian or Other Pacific Islander. Data on race and ethnicity were obtained from baseline interview responses, which asked participants to report “predominant race.” For our study, we collapsed the previous racial and ethnic categories into the following 4 groups: non-Hispanic White participants were classified as “White”; Hispanic White and Hispanic African American participants were classified as “Hispanic”; and non-Hispanic African American participants were classified as “Black.” Because of the small numbers, all other categories were classified as “other.”

The primary outcome of our study was sPTD, defined as delivery after preterm labor or preterm premature rupture of membranes at <34 weeks of gestation. The secondary outcomes included sPTD at <37 weeks of gestation and overall PTD (medically indicated and spontaneous) at <34 and <37 weeks of gestation.

The baseline demographics were compared among racial and ethnic groups using chi-square or Fisher exact tests for categorical variables and the Wilcoxon rank-sum test or *t* test for continuous variables, as appropriate. We fitted logistic regression models using White race as a reference to measure the differences in the rates of PTD (spontaneous and overall PTD at <34 and <37 weeks of gestation) among racial and ethnic groups while adjusting for confounders. We performed additional sensitivity analyses within each treatment group, using chi-square tests to compare the rates of PTD by race and ethnicity.

## Results

**Figure 1:**
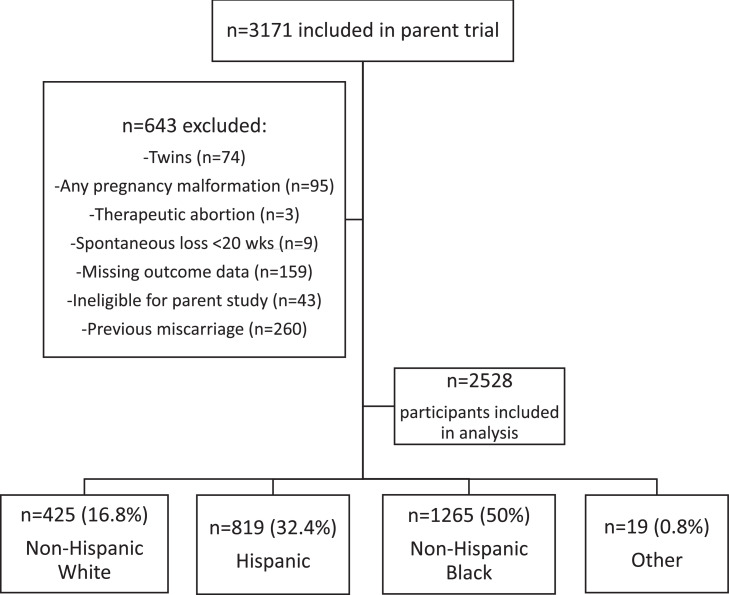
Population analysis The figure shows a flow diagram of study participants. A total of 643 participants in the parent trial were excluded. Of 2528 participants who were included, 425 were White, 819 were Hispanic, 1265 were Black, and 19 were other.

Of a total of 3171 participants included in the parent trial, 2528 individuals (79.7%) were included in our analysis. Excluded subjects are listed in Figure 1. Among the participants, 425 (16.8%) were non-Hispanic White, 819 (32.4%) were Hispanic, 1265 (50%) were Black, and 19 (0.8%) were not in one of those categories (Figure 1).

Kane. Low-dose aspirin for preterm delivery prevention by race. Am J Obstet Gynecol Glob Rep 2023.

The baseline characteristics are presented in Table 1. Treatment assignment (LDA vs placebo) and gestational age at randomization did not differ significantly among racial and ethnic groups (*P*=.87 and *P*=.24, respectively). All other baseline characteristics differed significantly, including maternal age, body mass index (BMI), tobacco and alcohol use, years of education, pregnancy loss, and marital status.

**Table 1:** Baseline characteristics of participants

Variable	White	Hispanic	Black	Other	*P* value
	n=425 (16.8)	n=819 (32.4)	n=1265 (50.04)	n=19 (0.75)	
Assigned aspirin	212 (49.9)	400 (48.8)	641 (50.7)	9 (47.4)	.87
Maternal age (y)	21 (19–27)	20 (18–23)	19 (17–21)	24 (20–28)	<.0001
AMA (>35)	8 (1.9)	8 (0.98)	1 (0.08)	1 (5.3)	<.0001
BMI (kg/m^2^)	22.2 (19.8–25.1)	23.5 (21.1–26.1)	22.4 (20.0–25.7)	21.5 (20.0–23.4)	<.0001
<18.5	40 (9.5)	91 (11.2)	149 (11.9)	3 (15.79)	.0005
18.5–24.9	273 (64.6)	464 (57.1)	745 (59.4)	13 (68.4)	
25.0–29.9	65 (15.4)	199 (24.5)	225 (17.9)	1 (5.3)	
>30.0	43 (10.2)	58 (7.1)	135 (10.8)	2 (10.5)	
Education (y)	12 (11–15)	10 (7–12)	12 (10–12)	12 (8–15)	<.0001
Married	177 (41.7)	253 (30.9)	58 (4.6)	7 (36.8)	<.0001
Tobacco use	130 (30.6)	13 (1.6)	113 (9.0)	1 (5.3)	<.0001
Alcohol use	38 (9.0)	3 (0.37)	27 (2.1)	0 (0)	<.0001
Gestational age at randomization (wk)	20.3 (17.3–23.1)	20.6 (17.3–23.6)	20.3 (17.6–23.0)	19.9 (17.4–25.0)	.2355

Categorical data are presented as number (percentage), and continuous data as presented as median (interquartile range). *P* values were calculated using the chi-square test for categorical data and the Wilcoxon test for continuous data.

*AMA*, advanced maternal age; *BMI*, body mass index.

The overall rate of sPTD at <34 weeks of gestation differed significantly among racial and ethnic groups, with the highest rates among Black individuals (2.8%) compared with White (1.2%) and Hispanic (1.2%) individuals (*P*=.041). A similar pattern was seen for sPTD at <37 weeks of gestation (Blacks, 8.5%; Hispanics, 6.2%; Whites, 4.9%; *P*=.009), overall PTD at <34 weeks of gestation (Blacks, 3.6%; Hispanics, 1.6%; Whites, 1.4%; *P*=.030), and overall PTD at <37 weeks of gestation (Blacks, 10%; Hispanics, 7.6%; Whites, 6.4%; *P*=.029).

Using White race as a reference, Black race was independently associated with sPTD at <37 weeks of gestation (odds ratio [OR], 1.75; 95% confidence interval [CI], 1.08–2.81; *P*=.02), overall PTD at <34 weeks of gestation (OR, 2.46; 95% CI, 1.07–5.64; *P*=.03), and overall PTD at <37 weeks of gestation (OR, 1.62; 95% CI, 1.06–2.49; *P*=.03) in our unadjusted analyses (Table 2). There was a 2-fold increase in sPTD at <34 weeks of gestation in Black participants, but this finding was not statistically significant (OR, 2.21; 95% CI, 0.89–5.46; *P*=.09) (Table 2).

**Table 2:** Rates of PTD among White, Hispanic, and Black racial and ethnic groups

Outcome	Race and ethnicity	OR (95% CI)	*P* value	aOR (95% CI)^a^	*P* value
sPTD at <37 wk	White	1.00 (reference)		1.00 (reference)	
	Hispanic	1.26 (0.75–2.11)	.38	1.25 (0.69–2.27)	.46
	Black	1.75 (1.08–2.81)	.02	1.56 (0.92–2.64)	.10
PTD at <37 wk	White	1.00 (reference)		1.00 (reference)	
	Hispanic	1.12 (0.75–1.90)	.45	1.14 (0.67–1.94)	.64
	Black	1.62 (1.06–2.49)	.03	1.38 (0.86–2.22)	.19
sPTD at <34 wk	White	1.00 (reference)		1.00 (reference)	
	Hispanic	0.99 (0.35–2.80)	.99	1.08 (0.35–3.30)	.89
	Black	2.21 (0.89–5.46)	.09	1.71 (0.67–4.40)	.26
PTD at <34 wk	White	1.00 (reference)		1.00 (reference)	
	Hispanic	1.08 (0.42–2.78)	.87	1.01 (0.37–2.79)	.99
	Black	2.46 (1.07–5.64)	.03	1.64 (0.69–3.89)	.26

*aOR*, adjusted odds ratio; *CI*, confidence interval; *OR*, odds ratio; *PTD*, preterm delivery; *sPTD*, spontaneous preterm delivery.

a The adjusted model controls for exposure to low-dose aspirin, maternal age, body mass index, tobacco and alcohol use, years of education, pregnancy loss, and marital status.

Maternal age, BMI, tobacco and alcohol use, years of education, pregnancy loss, and marital status differed significantly among the groups. After adjusting for the use of LDA and confounders that differed at baseline, Black race was no longer an independent risk factor for sPTD or overall PTD at <34 or <37 weeks of gestation (Table 2).

**Figure 2:**
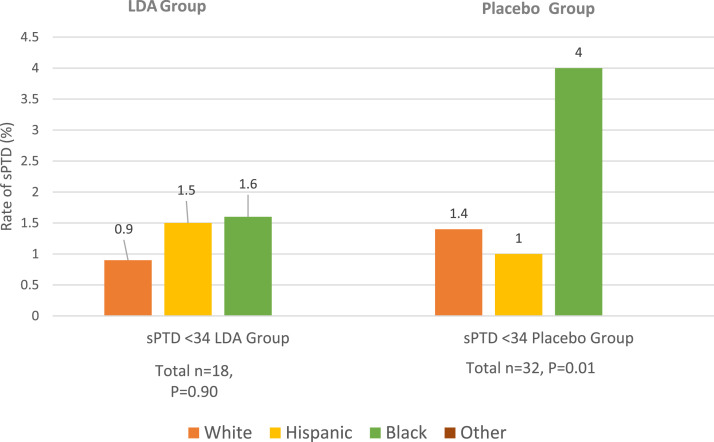
Rates of sPTD at <34 weeks of gestation by treatment assignment The graph shows the results of the sensitivity analyses within each treatment group comparing the rates of sPTD at <34 weeks of gestation by race. The rates of sPTD at <34 weeks of gestation differed significantly among racial and ethnic groups within the placebo group. There was no significant difference in the rates of sPTD in the LDA group. *LDA*, low-dose aspirin; *sPTD*, spontaneous preterm delivery.

We performed sensitivity analyses to assess the rate of PTD by treatment assignment. In the placebo group alone, the rates of sPTD at <34 weeks of gestation differed significantly by race (Blacks, 4%; Hispanics, 1.0%; Whites, 1.4%; *P*=.01). However, in the LDA group, the rates of sPTD at <34 weeks of gestation did not differ by race (Blacks, 1.6%; Hispanics, 1.5%; Whites, 0.9%; *P*=.90) (Figure 2). The analyses of the other outcomes in the placebo group revealed that there was a significant difference in the rates of overall PTD at <34 weeks of gestation (*P*=.01), a near-significant difference in sPTD at <37 weeks of gestation (*P*=.06), and a nonsignificant difference for overall PTD at <37 weeks of gestation (*P*=.19) (Supplemental Table 1). There was no significant difference in the rates of PTD within the LDA group for any of the measured outcomes: overall PTD at <34 weeks of gestation (*P*=.51), sPTD at <37 weeks of gestation (*P*=.31), or overall PTD at <37 weeks of gestation (*P*=.09) (Supplemental Table 1).

Kane. Low-dose aspirin for preterm delivery prevention by race. Am J Obstet Gynecol Glob Rep 2023.

## Discussion

### Principal findings

The Black race was associated with higher rates of sPTD and overall PTD at <34 and <37 weeks of gestation. However, the use of LDA mitigated racial disparities in the rates of sPTD in nulliparous individuals without comorbidities.

### Results in the context of what is known

LDA is gaining recognition as a potential safe and inexpensive intervention to prevent preterm birth.^22^ Efforts to eliminate the disparity in preterm birth incidence by race are challenged by the multifactorial nature of preterm birth and the need to obviate one of the underlying drivers of this disparity, structural racism. Our study demonstrates that the use of LDA can mitigate racial disparities in sPTD in this low-risk cohort. We found that Black participants experienced significantly higher rates of all measured PTD outcomes. We found that, when controlling for baseline demographics and the use of LDA, Black race was no longer an independent risk factor for PTD in the group that received LDA. Our novel findings warrant further study. Despite increasing evidence of efficacy,^15^^,^^16^^,^^18^, ^19^, ^20^ LDA is not currently recommended as a prevention strategy for preterm birth. Studying this intervention in a contemporary US cohort, with the possibility of reaffirming whether racial disparities in preterm birth can be mitigated, is of utmost importance.

Race is increasingly recognized as a sociopolitical construct with far-reaching effects.^23^^,^^24^ It is recognized that the association between Black race and clinical outcomes is not related to race but to the experience of racism associated with having Black skin. Sociodemographic factors account for some of the racial disparities in the rates of PTD: income, education, neighborhood, paternal acknowledgment, and maternal stress differ by race and have been shown to partially account for racial differences in the rates of PTD.^8^^,^^13^^,^^14^^,^^25^^,^^26^ However, racial disparities in birth outcomes persist even after controlling for education and socioeconomic status.^27^^,^^28^ There is a growing understanding that a history of discrimination can affect biology, including through inflammatory and cardiovascular pathways.^29^^,^^30^ Recent studies have shown that segregation and discrimination in early life lead to increased inflammatory markers, amplifying the inflammatory effects of later-life stressors.^31^ Exposure to discrimination has been associated specifically with adverse birth outcomes, including PTD^32^^,^^33^: there is evidence that such outcomes are mediated by specific biological pathways affected by discrimination, including epigenetic factors, such as telomere shortening^34^ or immune system function.^35^ Such findings underscore the concept that anti-Black racism rather than race itself is a driving force behind the observed racial inequities in birth outcomes in the United States.^7^^,^^36^

The biological consequences of racism may explain differing etiologies of PTD by race. Black individuals with a history of PTD are more likely to have pregnancies complicated by maternal stress and cervical insufficiency, as opposed to decidual hemorrhage.^25^ Biomarkers associated with PTD, such as interleukin 1 or tumor necrosis factor-alpha, have been found to be significantly different among races, providing further evidence of varying phenotypes predisposing to PTD.^37^^,^^38^ Of note, 1 large study examining inflammatory biomarkers to predict sPTD found little overlap between White and Black groups and concluded that the optimal models were race dependent.^38^ Biomarkers for PTD seem to contribute to the differential risk of PTD depending on an individual's race.^37^ Racial differences in vaginal microbiota have been documented,^39^^,^^40^ and there are some evidences that a more diverse microbiome seen in some African American populations may predispose to preterm birth.^6^^,^^41^ Moreover, differences in levels of polymorphisms in inflammatory response genes have been associated with the risk of PTD and have been shown to differ between White and Black people.^42^

Overall, these data support the claim that there are different underlying etiologies of PTD for Black individuals compared with White individuals; these differences are likely due to a complex interplay between environmental factors and their effect on biology.^7^^,^^43^ LDA, which acts on both inflammatory and ischemic pathways,^44^ may mitigate racial disparities in PTD by targeting specific dysregulated pathways more likely to lead to PTD in Black individuals.

### Clinical implications

PTD is a long-standing challenge in the field of obstetrics. The growing recognition of racial disparities in maternal and fetal health outcomes, and especially the disproportionate burden of PTD on Black women,^6^^,^^45^ has refocused research and initiatives toward addressing this discrepancy and the structural racism at the core of these disparities. Our current study suggests that LDA may be a promising means to mitigate these sociopolitically rooted disparities that require further study.

In addition to its effects to reduce sPTD,^15^^,^^16^^,^^18^, ^19^, ^20^ LDA is known to prevent adverse events in pregnancy for certain populations.^22^ Based on the results of a large 2014 meta-analysis, LDA is currently recommended by the US Preventive Services Task Force to prevent preeclampsia in high-risk women.^46^ In addition, LDA has been shown to reduce the risk of fetal growth restriction, especially for those at risk of preeclampsia.^47^^,^^48^ Furthermore, there is evidence that LDA has differential effects by race on these birth outcomes, including spontaneous abortions and preeclampsia.^49^^,^^50^ Such data demonstrate the importance of further research into the effect of aspirin on birth outcomes by race and ethnicity.

### Research implications

Establishing the pathway through which LDA might mitigate racial disparities in rates of PTD can help to better target this intervention to populations that may most benefit. Although genetic pathways have been implicated as a potential contributor to these disparities, there is increasing recognition that race is a social construct and merely a proxy for social and environmental stressors that disproportionately affect birth outcomes among groups marginalized by their race or ethnicity in the United States. Elucidating the factors that most affect the rates of PTD in Black populations will allow for targeted clinical approaches.

As our study found that LDA's effects in mitigating disparities in PTD are more pronounced in sPTD and overall PTD at <34 weeks of gestation than in sPTD and overall PTD at <37 weeks of gestation, future studies should also focus on possible mechanisms by which LDA may preferentially affect very early PTD in specific populations.

### Strengths and limitations

Our study is limited by inherent challenges in artificial racial and ethnic categorization, including the inability to represent the overlap between Hispanic ethnicity and other racial categories. Because these categorizations, even with self-identification, are somewhat arbitrary social designations, we employed them in their initial context. Furthermore, this format of racial and ethnic categorization follows the standards of the official data on preterm birth currently distributed by the CDC.^11^ In addition, this study is a secondary analysis of a randomized trial. However, this study was not specifically designed to address our research question. However, given the wide availability of over-the-counter aspirin, it is challenging to study LDA outside of a randomized trial because of the difficulty of completely ascertaining treatment exposure. Our results are only generalizable to low-risk nulliparous individuals without comorbidities, as this was the population included in the trial. Nonetheless, this may be advantageous in this case, as most current interventions are targeted at parturients with previous obstetrical complications.

An important consideration for this analysis is that participants were recruited from 1989 to 1991. Sociodemographic factors affecting preterm birth by race at that time may differ from such factors today. However, the racial disparity in preterm birth has remained relatively stable since that time; for example, in 1990, the CDC reported rates of PTD of 18.9% for non-Hispanic Black, 8.4% for non-Hispanic White, and 11.0% for Hispanic individuals.^51^ In 2020, the rates were 14.4%, 9.1%, and 10%, respectively.^11^ A recent temporal trends analysis of preterm birth from 1971 to 2018 found that the cumulative incidence of preterm birth over this 50-year period demonstrates a similar Black-White disparity (17.6% vs 9.6%).^9^ In addition, more contemporary data show the persistence in hospital settings of structural racism.^52^, ^53^, ^54^ Such persistent disparities indicate that driving factors for preterm birth in such populations are likely stable and have not been adequately addressed over the last 30 years, further highlighting the need for novel interventions to target this disparity.

There are several strengths of this study, including that data were collected from a large RCT with data obtained granularly by trained research staff. There is complete ascertainment of treatment assignment. The data were collected at clinical centers across the country, which represent geographic, ethnic, and racial diversity.

### Conclusions

Our study shows that the use of LDA mitigated racial disparities in sPTD at <34 weeks of gestation in nulliparous individuals without comorbidities. More research is needed to explore whether this finding can contribute to mitigating the long-standing disparity in the rates of PTD by race and ethnicity in the United States. Further investigation may allow us to understand whether this is true for individuals at higher risk of pregnancy complications. Clarifying the mechanism through which LDA exerts this effect may allow more targeted interventions to protect against PTD. Given the significant burden of PTD for Black individuals in the United States, our findings represent a promising area of research to promote more equitable maternal and fetal health outcomes.

## Appendix Supplementary materials

Image, application 1

## Footnotes

Supplementary material associated with this article can be found, in the online version at doi:10.1016/j.xagr.2023.100273.
