# Supplementary material for: Low-dose aspirin and racial disparities in spontaneous preterm delivery in low-risk individuals
Source: AJOG Glob Rep. 2023 Oct 5;3(4):100273. doi: 10.1016/j.xagr.2023.100273 (PMC10682009; doi:10.1016/j.xagr.2023.100273)
Supplement: Supplementary file 1 [file mmc1.docx]

**SUPPLEMENTAL TABLE 1. Rates of PTD by treatment assignment group.**

|  | White (%) | Hispanic (%) | Black (%) | Other (%) | P-value | N |
| --- | --- | --- | --- | --- | --- | --- |
| sPTD <34 LDA | 0.94 | 1.50 | 1.56 | 0 | 0.90 | 18 |
| sPTD <34 Placebo | 1.41 | 0.95 | 4.01 | 0 | 0.01 | 32 |
|  |  |  |  |  |  |  |
| PTD <34 LDA | 1.42 | 2 | 1.51 | 0 | 0.52 | 30 |
| PTD <34 Placebo | 1.41 | 1.19 | 4.33 | 0 | 0.01 | 35 |
|  |  |  |  |  |  |  |
| PTD <37 LDA | 4.72 | 8.5 | 9.98 | 0 | 0.09 | 108 |
| PTD <37 Placebo | 7.98 | 6.68 | 10.1 | 0 | 0.19 | 108 |
|  |  |  |  |  |  |  |
| sPTD <37 LDA | 4.25 | 7 | 7.64 | 0 | 0.31 | 86 |
| sPTD <37 Placebo | 5.63 | 5.49 | 9.29 | 0 | 0.06 | 93 |

P-values were determined by chi-square test.

**Abbreviations:** LDA, Low Dose Aspirin; PTD, preterm delivery; sPTD, spontaneous preterm delivery.
